# Tensile Property and Corrosion Performance of Ag Microalloying of Al-Cu Alloys for Positive Electrode Current Collectors of Li-Ion Batteries

**DOI:** 10.3390/ma15155126

**Published:** 2022-07-23

**Authors:** Zixuan Peng, Dongyan Ding, Wenlong Zhang, Yongjin Gao, Guozhen Chen, Yonglin Xie, Yongqi Liao

**Affiliations:** 1 School of Materials Science and Engineering, Shanghai Jiao Tong University, Shanghai 200240, China; pengzixuan@sjtu.edu.cn (Z.P.); zhangwl@sjtu.edu.cn (W.Z.); 2 SJTU-Huafon Joint Lab, Shanghai Huafon Al Co., Ltd., Shanghai 201506, China; gao.yongjin@huafeng.com (Y.G.); chen.guozhen@huafeng.com (G.C.); xie.yonglin@huafeng.com (Y.X.); liao.yongqi@huafeng.com (Y.L.)

**Keywords:** electrode materials, Al-Cu-Ag alloys, microscopic structure, tensile properties, corrosion performance

## Abstract

The development of a current collector for Li-ion batteries is of great significance for improving the performance of Li-ion batteries. Tensile property and corrosion performance of the positive electrode current collectors are an indispensable prerequisite for the realization of high-performance Li-ion batteries. In our study, the effects of Ag alloying on the microscopic structure, electrical conductivity, tensile property and corrosion resistance of Al-xCu (x = 0.1–0.15%) alloy foils were investigated. Moderate Ag addition on the Al-Cu alloy could reduce the size of second phases and promote the formation of second phases. The tensile strength of the Al-0.1Cu-0.1Ag alloy was higher than that of the Al-0.1Cu alloy at both room and high temperatures. All of the alloy foils demonstrated high electrical conductivity around 58% ICAS. The corrosion potential and corrosion current density of the Al-0.1Cu alloy were demonstrated by Tafel polarization to be −873 mV and 37.12 μA/cm^2^, respectively. However, the Al-0.1Cu-0.1Ag alloy showed enhanced corrosion resistance after the Ag element was added to the Al-0.1Cu alloy, and the Al-0.1Cu-0.1Ag alloy had a greater positive corrosion potential of −721 mV and a lower corrosion current density of 1.52 μA/cm^2^, which suggests that the Ag element could significantly improve the corrosion resistance of the Al-Cu alloy.

## 1. Introduction

With the over-exploitation and use of fossil fuels by humans, ecological environmental problems have become increasingly prominent. Economic and social development is inseparable from the promotion of energy. However, oil, natural gas, and coal are examples of non-renewable energy sources which not only face exhaustion problems, but also cause serious environmental damage [1].

One of the keys to solving the current problems of fossil energy exhaustion and environmental pollution lies in energy storage components, which can effectively use existing energy sources and develop new energy sources [2,3,4]. As a new type of energy storage device with relatively mature research and application, lithium-ion batteries (LIBs) have many advantages such as high-power density, good charge/discharge stability, and long cycle life performance [5,6]. Portable electronics, electric automobiles, and hybrid electric vehicles all frequently use LIBs. Furthermore, LIBs are considered to be the most promising energy storage system which can reduce our dependence on petroleum, emissions of greenhouse gases, and criteria pollutants including nitrogen oxide, carbon monoxide, sulfur oxide and hydrocarbon [7,8,9,10].

In the electrochemical applications of LIBs, the current collector is a crucial component that can transfer active slurry, collect electrons, and transport electrons. [11]. For example, the improvement of conductivity and corrosion resistance of the current collector contributes to the improvement of capacity, charge and discharge efficiency, and cycle stability of LIBs [12]. Aluminum alloys are widely employed as current collectors in LIBs due to their excellent conductive properties. Additionally, the electrolyte does not readily erode the aluminum foil’s passivation layer during the charge and discharge operation. On the contrary, copper is easily oxidized at a higher potential. Thus, it is employed as a negative electrode current collector. Seung-Taek et al. [13] reported that Al is the material of choice for high-voltage LIBs and it can be protected by a thin and dense oxide passive layer, Al_2_O_3_. At the same time, aluminum foil has high conductivity and stable electrochemical characteristics. These advantages make it widely used in current collectors for lithium-ion battery cathodes. Moreover, different microalloying elements have different effects on the physical and electrochemical properties of the current collector. Xu et al. [14] discovered that La-containing Al-Fe-Cu alloy had good electrical conductivity, tensile property and corrosion resistance compared with La-free Al-Fe-Cu alloy. Zhu et al. [15] found that adding a reasonable amount of La to Al-Fe-Mn alloy refined the grains and decreased the amount of precipitates at the grain boundaries, which could increase the alloy’s strength.

Owing to their ideal combination of high specific strength, outstanding fatigue properties and superior heat resistance, Al-Cu binary alloys have shown great potential in aircraft and automobile manufacturing industries [16,17,18]. According to previous studies, there are two main different precipitates in Al-Cu alloys. The body-centered tetragonal θ’(Al_2_Cu) phase is the main strengthening phase in the Al-Cu binary system [19]. The other precipitation is the θ”(Al_3_Cu) phase, which is also tetragonal in structure and three layers of Al atoms distribute between two layers of Cu atoms from (001)_Al_ faces [20]. Additionally, it was reported that temperature could affect the phase transformation of Al-Cu alloys. At high temperature, the precipitation process, i.e., G.P. zone→ θ” phase→ θ’ phase, could be enhanced [21]. Gao et al. [22] reported that θ’ precipitation in an Al-Cu thin-film alloy could boost the interconnects’ electromigration lifetime and raise the alloy’s strength thanks to a stronger bond net. The Al-Ag binary alloys, on the other hand, are mostly utilized as diverse electrical contacts for application in plates or wire [23]. In order to improve the alloys’ thermal stability, stress-corrosion cracking and mechanical properties, a moderate amount of Ag addition could have a significant impact [24]. Jafarian et al. [25] reported that in the ultrafine grains of Al-Ag alloys, Al-Ag precipitates occurred, and it was clear that these precipitates had a beneficial effect on strengthening the mechanical properties of alloys.

Many studies have been conducted on Al-Cu alloying systems, while some works focused on the microstructure of Al-Cu-Ag alloys. In Al-Cu-Ag alloys, there are two primary phases: the θ’(Al_2_Cu) phase and γ’ (AlAg_2_), with the θ’ phase as the main strengthening phase. Senninger et al. [26] found that the addition of Ag had little influence on the precipitation of θ’ in the Al-Cu alloy, which indicates that Ag addition had a minor impact on the mechanical characteristics of the Al-Cu alloy. Genau et al. [27] reported that two different orientation relationships had been observed between Al_2_Cu and Ag_2_Al. Hecht et al. [28] reported that in bulk, polycrystalline samples, lamellar eutectic (Al)/Al_2_Cu from ternary Al-Cu-Ag liquid alloys demonstrated quite remarkable properties. Zhou et al. [29] reported that in unconstrained grown eutectics, Al_2_Cu phases exhibited significant intermetallic anisotropy and elongated morphology, while the Ag_2_Al phases had a regular morphology in eutectic.

Thus far, only a few studies have been published on the mechanical characteristics and corrosion performance of Al-Cu-Ag alloy foils used as the current collector of LIBs. In this study, we explore the influence of Ag content on the microstructure, mechanical characteristics, electrical properties, and corrosion resistance of Al-Cu-Ag alloys.

## 2. Materials and Methods

The aluminum alloys of Al-0.1Cu, Al-0.1Cu-0.08Ag, Al-0.1Cu-0.1Ag and Al-0.15Cu-0.1Ag were fabricated using pure aluminum ingot and Al-50wt.% Cu master alloys. After inductive and casting, the homogenization at 590 °C for 8 h was conducted for the aluminum alloy ingots. After the homogenization treatment, the ingots were hot rolled into a thickness of 3 mm and air cooled to room temperature. Finally, a thickness of 0.07–0.09 mm of alloy foils was achieved by cold rolling the hot-rolled plates. During the production process of LIBs, slurry containing active materials will be coated on the surface of the current collector. After the coating process, the coated current collector needs to be dried at high temperature to remove the N-methylpyrrolidone (NMP) solvent [30]. To imitate the drying process, the cold-rolled foils were annealed at 125 °C for 5 h.

The longitudinal section grain structure of the as-homogenized alloy foils was studied under polarized light using an optical microscope (OM, ZEISS imageA1m, Jena, Germany). A scanning electron microscope was used to examine the microstructure and second phase particles of the homogenized samples and foils (NOVA NANOSEM 230, FEI, Hills-borough, OR, USA) operating at 5 KV, and energy dispersive spectroscopy (EDS) operating at 15 KV. The samples for microstructural observation were first mechanically wet ground with 500, 1000, 2000, 3000 and 5000 mesh SiC sandpapers, and then polished on the cashmere polishing clothes with diamond spray (with a particle size of 0.25 µm).

Mechanical properties were characterized through tensile testing using a Zwick/Roell Z020 (Ulm, Germany) universal tension machine at 25. The high temperature tensile tests were carried out with electromechanical universal testing machine (CMT 5105, Meters Testing Machine Factory, Tianjin, China). The testing was conducted according to the standard of ASTM E8/E8M-13a at a tensile speed of 1 mm/min. At least four parallel specimens were taken for each alloy to guarantee accuracy.

Tensile testing was performed at 25 °C on a Zwick/Roell Z020 universal tension machine (Ulm, Germany). The electromechanical universal testing machine was used to perform the high temperature tensile tests (CMT 5105, Meters Testing Machine Factory, China). The tests were carried out in accordance with ASTM E8/E8M-13a standards at a tensile speed of 1 mm/min. To ensure accuracy, at least four parallel specimens of each alloy were collected.

Corrosion resistance of the foils was characterized through the Tafel polarization curve testing in a CHI 660C (Chenhua Instrument Company, Shanghai, China) electrochemical device. All the samples were washed with acetone, absolute ethanol and deionized water in an ultrasonic wave cleaner for about 10 min, respectively. Finally, a three-electrode Tafel polarization curve test was performed. The sample, saturated calomel electrode, and platinum served as the working, reference, and auxiliary electrodes in the three-electrode system, respectively. Each alloy had three parallel samples to assure measurement accuracy. The electrochemical test used a 3.5 wt.% NaCl solution as the electrolyte. All electrochemical experiments were carried out at 25 °C with a scanning speed of 1 mV/s and a range of −1 V to 0 V. Before starting to test, to ensure stability of the system, the assembled three-electrodes system needed to stand for sufficient time. In this work, the standard is that the fluctuation of the open circuit voltage was within the range of 0.01 V. After the test, extrapolation was used to draw the tangent of the anode and cathode curves. The abscissa corresponding to the intersection point was the corrosion potential, and the ordinate was the corrosion current density. A scanning electron microscope was used to examine the corrosion surface of samples (NOVA NANOSEM 230, FEI, Hillsborough, OR, USA).

## 3. Results and Discussion

### 3.1. Microstructure

**Figure 1:**
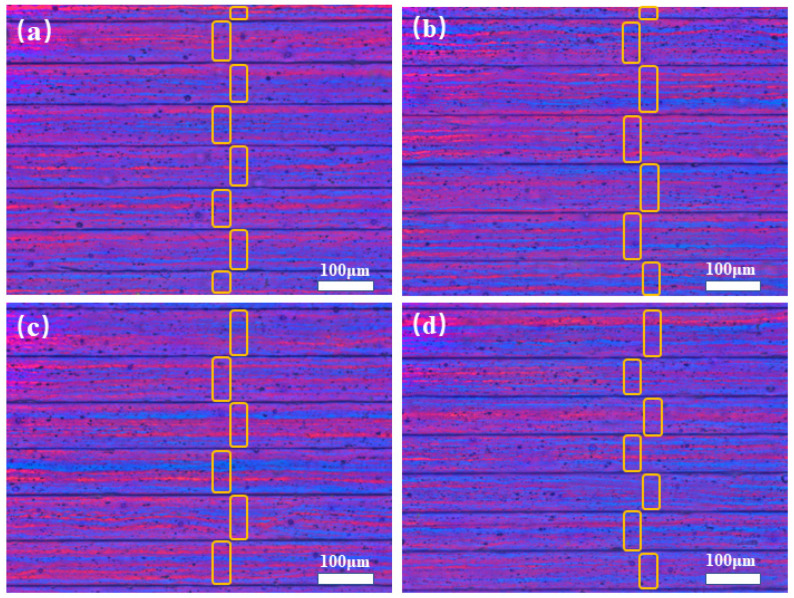
OM images of longitudinal sections of four heat-treated alloy foils: (**a**) Al-0.1Cu alloy, (**b**) Al-0.1Cu-0.08Ag alloy, (**c**) Al-0.1Cu-0.1Ag alloy, (**d**) Al-0.15Cu-0.1Ag alloy.

Figure 1 shows the longitudinal section microscopic structure of the Al-0.1Cu, Al-0.1Cu-0.08Ag, Al-0.1Cu-0.1Ag and Al-0.15Cu-0.1Ag alloys annealed at 125 °C for 5 h. The rolling texture along the rolling direction of the alloy foil was demonstrated through anodic coating and observed in the polarized light mode of the metallographic micro-scope. We folded the alloy foil in half several times to form stacked foils before preparing the embedded samples for anodic coating. As shown below, each box represents a foil. There are between six and seven layers in each image. The rolling tension causes the grains to extend along the rolling direction, resulting in a slender fibrous structure rather than typical annealing grains. Meanwhile, both textures and grains were observed together in these four alloys (Figure 1a–d), and the addition of trace Ag caused a slight increase in grain size (Figure 1a–c). It was found that when the mass fraction of Cu increased from 0.1 wt.% to 0.15 wt.%, the grain size distribution of the Al-0.15Cu-0.1Ag alloy was more uniform and the grain size became finer (Figure 1c,d).

**Figure 2:**
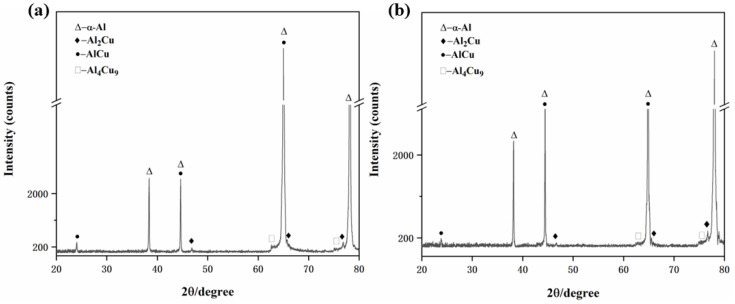
X-ray diffraction (XRD) patterns of the heat-treated alloy foils: (**a**) the Al-0.1Cu alloy, (**b**) the Al-0.1Cu-0.1Ag alloy.

Figure 2 shows X-ray diffraction patterns of heat-treated Al-0.1Cu and Al-0.1Cu-0.1Ag alloy foils. When the Ag content increased from 0 wt.% to 0.1 wt.%, The second phase precipitation types in the Al-0.1Cu-yAg (y = 0, 0.1 wt.%) alloys did not differ appreciably, which indicates that the Ag element had little effect on the precipitation of AlCu phases.

**Figure 3:**
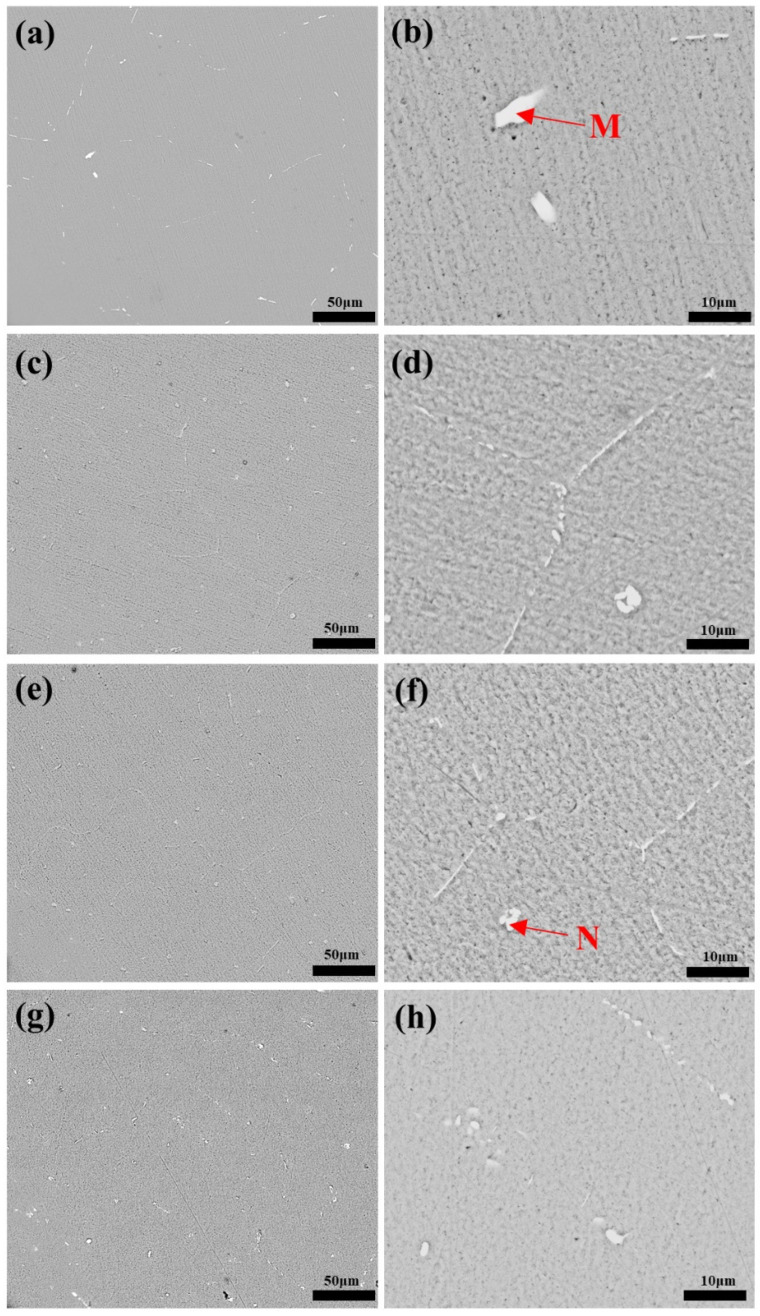
Images of four heat-treated alloy foils obtained using SEM/BSE: (**a**,**b**) Al-0.1Cu alloy, (**c**,**d**) Al-0.1Cu-0.08Ag alloy, (**e**,**f**) Al-0.1Cu-0.1Ag alloy, (**g**,**h**) Al-0.15Cu-0.1Ag alloy.

In order to explore the influence of the content of Cu and Ag on the number and size of the precipitates of the alloys, SEM observation with backscattered electron (BSE) mode was conducted. Figure 3 shows BSE images of the Al-0.1Cu, Al-0.1Cu-0.08Ag, Al-0.1Cu-0.1Ag and Al-0.15Cu-0.1Ag alloy homogenized at 590 °C for 8 h. The dark background represents the Al matrix while the bright particles represent precipitates. As shown in Figure 3a,c,e,g, it can be seen that, near grain boundaries, the residual precipitates after the homogenization treatment tended to distribute discontinuously. The size, quantity and distribution of precipitates also varied. Through high-magnification observation, it was found that most of the precipitates were rod-like. When the addition of Ag increased to 0.08 wt.% (Figure 1b, Al-0.1Cu-0.08Ag), the number of precipitates along the grain boundaries increased significantly while more small-size precipitates appeared and aggregated together, indicating that Ag greatly changed the shape and distribution of precipitates. When the addition of Ag increased to 0.1 wt.% (Figure 3c, Al-0.1Cu-0.1Ag), the quantity of precipitates of the Al-0.1Cu-0.1Ag alloy increased but further aggregated. Furthermore, compared to the Al-0.1Cu-0.08Ag alloy, the Al-0.1Cu-0.1Ag alloy had more precipitates, which showed that the Ag element could promote the formation of the precipitates but restrained the growth of the precipitates. However, when the addition of Cu increased to 0.15 wt.% in Al-xCu-0.1Ag (x = 0.1, 0.15 wt.%) alloys (Figure 3e,g), in comparison with the Al-0.1Cu-0.1Ag alloy, the number of precipitates segregated at the grain boundaries of the Al-0.15Cu-0.1Ag alloy reduced.

**Figure 4:**
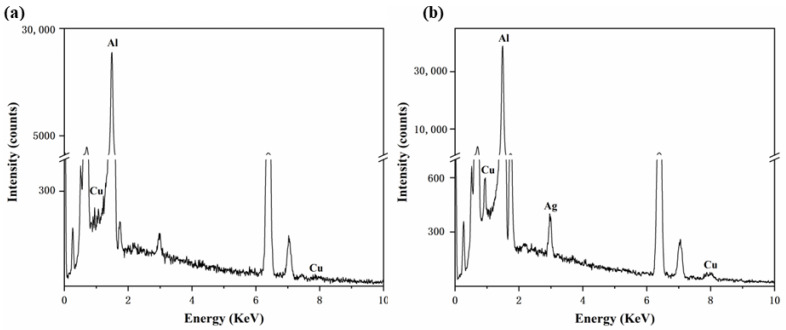
Typical EDS patterns of the (**a**) AlCu phase (M) shown in Figure 3b, (**b**) AlCuAg phase (N) shown in Figure 3f.

Energy dispersive spectroscopy (EDS) was used to determine the composition of the precipitates in the Al-xCu-yAg alloys. Figure 4a,b shows the EDS analyses of the precipitates labeled as M and N. It was found that the rod-shaped precipitate (M) was the AlCu phase, and the clustered rod-shaped precipitate (N) was the AlCuAg phase.

### 3.2. Mechanical Properties

In practice, LIBs usually operate in a temperature below 60 °C. Thus, the tensile properties of the four kinds of alloys were tested at room temperature and high temperature, corresponding testing results are shown in Table 1. At room temperature, the Al-0.1Cu alloy’s 0.2% offset yield stress (σ_0.2_), ultimate tensile stress (σ_b_) and the elongation (δ) were 175 ± 4 MPa, 189 ± 3 MPa and 1.6 ± 0.1%, respectively. When the testing temperature increased to 60 °C, σ_0.2_ and σ_b_ of the Al-0.1Cu alloy decreased to 156 ± 2 MPa and 176 ± 3 MPa, whereas δ increased to 1.7 ± 0.1%. With increasing temperature, there was a general tendency in all four experimental alloys towards a decrease in the tensile strength and increase in elongation. Such a change of strength and plasticity could be attributed to the movement of the dislocation dominated by the thermal activation of the cross slip, which lead to a decrease in strength and an increase in elongation [31].

A few early studies showed that isothermal aging could result in the precipitation of θ’, θ” and γ’ (AlAg_2_) precipitates for compositions of around 1 at. % Cu, 1 at. % Ag in the Al-Cu-Ag alloys [32]. In Al-Cu-Ag alloys, the main strengthening phases were θ” phases. There are two different factors that work together to affect the tensile strength of Al-Cu-Ag alloy. On the one hand, Ag-containing Al-Cu alloys may segregate the θ’ and Al-matrix interfaces, forming an Ag-rich double layers, which hindered or prevented the growth of the θ’ precipitates. At the same time, the addition of trace Ag may decrease the density of θ’ and θ” precipitates, which deteriorated the tensile properties of the alloys [33,34]. On the other hand, the addition of an appropriate amount of Ag can cause θ’ precipitates to distribute in a more uniform and finer manner, and the Ag element enhances the lattice vacancy density, reduces the interfacial energy, and provides additional formation for precipitation [35]. When the Ag content increased from 0% to 0.08% in Al-0.1Cu-xAg alloys at room temperature, the σ_0.2_ decreased from 175 ± 4 MPa to 170 ± 5 MPa and the σ_b_ from 189 ± 3MPa to 184 ± 5 MPa. When the Ag content further increased to 0.1%, the σ_0_._2_ increased to 179 ± 3 MPa and the σ_b_ to 196 ± 1 MPa. The addition of a moderate amount of the Ag element to the Al-0.1Cu alloy clearly had a weak strengthening effect.

### 3.3. Electrical Conductivity

An excess of precipitation may improve the pinning effect of the second phase while also causing certain negative effects such as a reduction in the alloy’s electrical conductivity according to previous studies [36,37,38]. The electrical conductivity testing results of the four annealed alloy foils are shown in Table 2. The addition of a trace amount of Ag to the Al-0.1Cu alloy resulted in a slight decrease in electrical conductivity. As the Ag content increased, the electrical conductivity of Al-0.1Cu-yAg alloy slightly decreased. However, the electrical conductivity values of all alloy foils were around 58% IACS (International Annealed Copper Standard), which can meet the application requirements. According to Figure 3, Ag addition on the Al-Cu alloy promoted precipitation of the second phases at the grain boundary and more small-size precipitates appeared, which may be the reason for the slight decrease in electrical conductivity.

### 3.4. Electrochemical Performance

**Figure 5:**
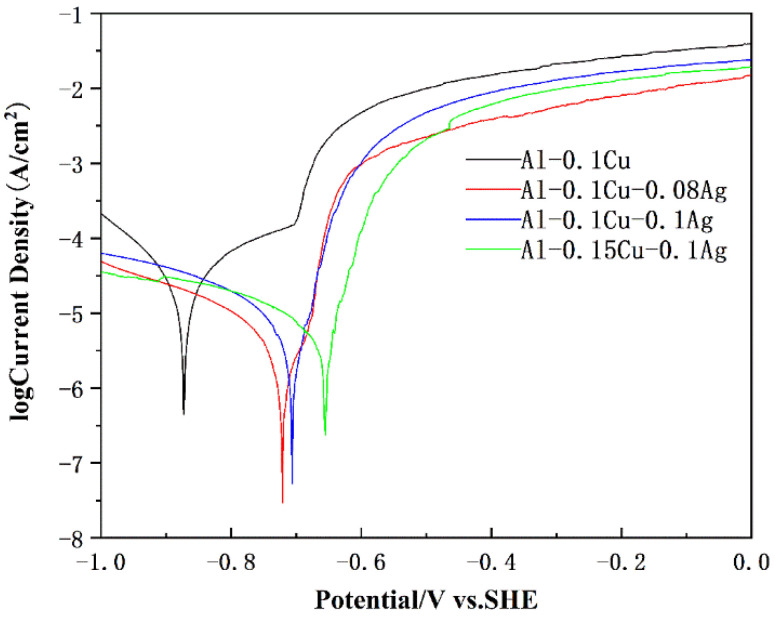
Typical Tafel polarization curves of the heat-treated alloy foils.

Tafel polarization curve studies were performed to investigate the electrochemical corrosion behavior of the alloys. Figure 5 shows typical Tafel curves of the four kind of alloy foils tested in 3.5 wt.% NaCl solution. The corrosion potential (E_corr_) and corrosion current (I_corr_) of each alloy could be calculated by extrapolating the Tafel curves of different alloys. Table 3 shows E_corr_ and I_corr_ corresponding to the Tafel curves. Each alloy was measured three times to ensure the accuracy of the results. The standard deviations of corrosion potential of four kinds of alloys were ±7 mV, ±5 mV, ±5 mV and ±6 mV, which could reflect the difference in corrosion resistance. However, the standard deviation of corrosion current density was too large to reflect it.

The corrosion potential of the Al-0.1Cu alloy, Al-0.1Cu-0.08Ag alloy, and Al-0.1Cu-0.1Ag alloy were −873 mV, −721 mV and −706 mV, respectively. This demonstrates that the addition of Ag could significantly increase the corrosion potential of the alloys. With the addition of Ag from 0 wt.% to 0.1 wt.%, the I_corr_ of the Al-Cu-xAg (x = 0, 0.08, 0.1 wt.%) alloys decreased from 37.12 μA to 5.49 μA. The E_corr_ indicates the corrosion thermodynamic of the material and the I_corr_ represents the corrosion kinetic characteristics of the alloy [39].

Generally speaking, higher E_corr_ means better chemical stability, and lower I_corr_ means a low rate of corrosion [40]. The inclusion of trace Ag increased the protection against corrosion of the alloys substantially. In addition, it was particularly noted that, compared to the Al-0.1Cu-0.1Ag alloy, the corrosion potential of the Al-0.15Cu-0.1Ag alloy further shifted positively from −706 mV to −655 mV. It is possible that the addition of Cu enhanced the alloy’s corrosion resistance, although the I_corr_ may have increased somewhat.

**Figure 6:**
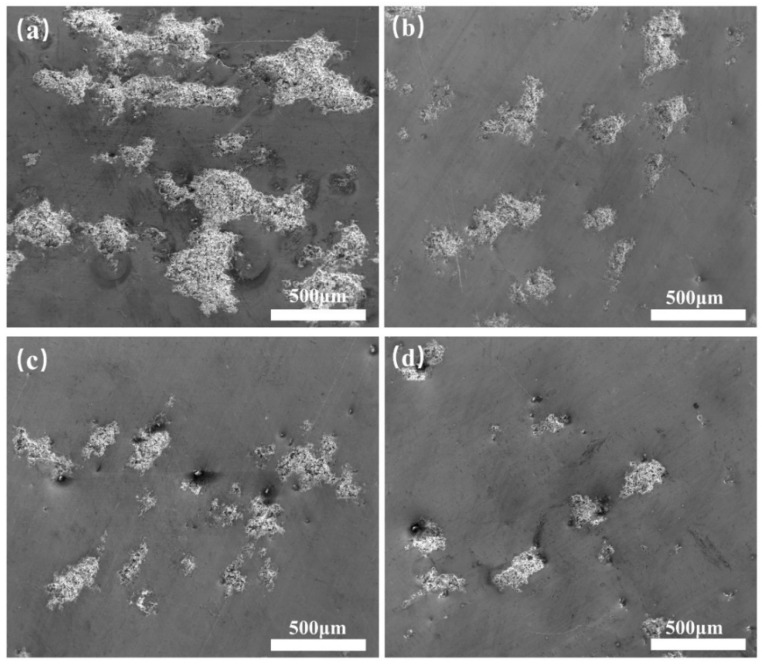
Images of four heat-treated alloy foils obtained using SEM/BSE: (**a**) Al-0.1Cu, (**b**) Al-0.1Cu-0.08Ag, (**c**) Al-0.1Cu-0.1Ag and (**d**) Al-0.15Cu-0.1Ag.

Due to the difference in the corrosion potentials of Cu between Ag-containing intermetallic phases, the corrosion pits were most likely to start from the precipitates, leading to the first corrosion pits of the Al matrix near the Cu or Ag-containing second phase particles. The corrosion surfaces of the four alloys tested after Tafel tests are shown in Figure 6. Comparing Figure 6a–c, the number and size of corrosion pits differed significantly among the Al-0.1Cu alloy, Al-0.1Cu-0.08Ag alloy, and Al-0.1Cu-0.1Ag alloy. After the Ag element was added, the number of corrosion pits decreased and the average size of the corrosion pits also decreased. This result indicates that the Ag element may greatly increase the alloy’s corrosion resistance. Meanwhile, Figure 6c,d showed the corrosion pits of the Al-0.1Cu-0.1Ag alloy and the Al-0.15Cu-0.1Ag alloy, respectively. It can be seen that when the Cu content increased from 0.1 wt.% to 0.15 wt.%, the quantity of the corrosion pits reduced and there was no significant change in the size of the corrosion pits, which also indicated that the increase in Cu content could improve the corrosion resistance of the alloy. These results were consistent with the parameters mentioned above.

**Figure 7:**
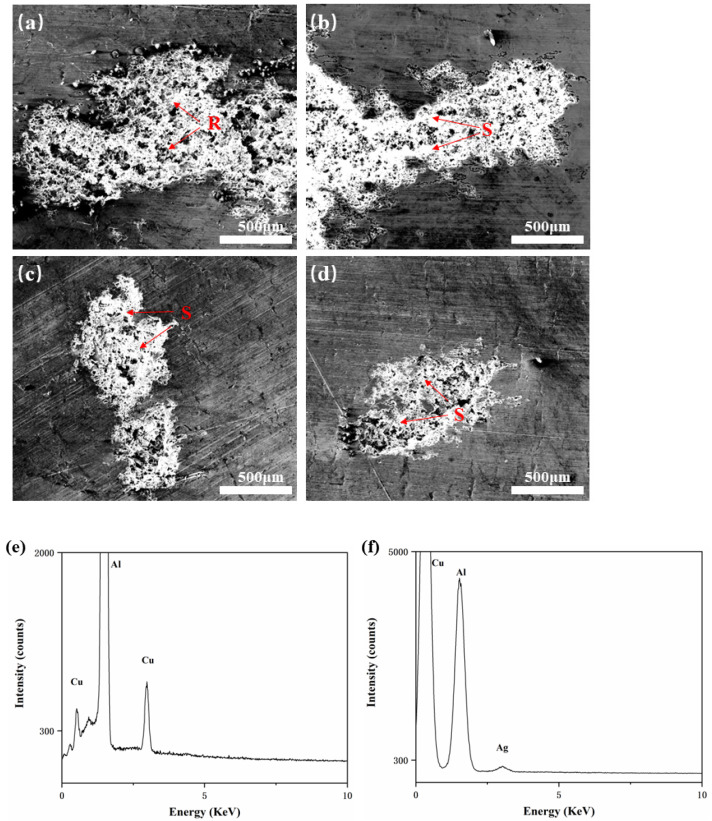
Corrosion surface morphology of four kinds of foils: (**a**) Al-0.1Cu alloy, (**b**) Al-0.1Cu-0.08Ag alloy, (**c**) Al-0.1Cu-0.1Ag alloy, (**d**) Al-0.15Cu-0.1Ag alloy, and (**e**,**f**) EDS patterns of corrosion regions R and S.

Figure 7 shows SEM photographs of the corroded area at high magnification. In order to analyze the residual substances in corrosion pits at regions R and S, EDS analyses were conducted. Using XRD analysis in conjunction, we were able to determine that the leftover component in region R was AlCu phase (Figure 7e). The presence of Cu-containing particles had a detrimental effect on corrosion resistance. The Cu-rich phase was the cathodic reaction’s catalyst and the pit’s nucleation location in comparison with the Al matrix, therefore, the corrosion would first occur around the AlCu phase [41]. However, after adding a trace amount of the Ag element to the Al-0.1Cu alloy, the E_corr_ of the alloy was shifted positively, the I_corr_ was greatly reduced, and the alloy’s corrosion resistance was significantly increased (Table 3). At the same time, it could be found that the residual substance at region S (Figure 7f) was composed of three elements: Al, Cu and Ag. This indicated that the addition of Ag may minimize the potential difference between the AlCu phase and the corrosion matrix, thereby reducing the tendency of alloy corrosion.

**Figure 8:**
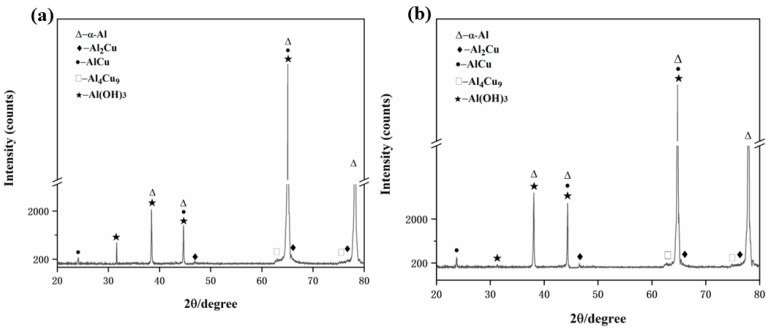
XRD patterns of the corrosion surfaces of the alloy foils tested: (**a**) the Al-0.1Cu alloy, (**b**) the Al-0.1Cu-0.1Ag alloy.

In order to explore the second phases of aluminum alloy after corrosion, we conducted XRD analyses of the Al-0.1Cu and Al-0.1Cu-0.1Ag alloy foils after corrosion, and Figure 8 shows the corresponding patterns. By comparing Figure 8a with Figure 2a, we found that the Al(OH)_3_ phase formed after the Al-0.1Cu alloy was electrochemically corroded in 3.5 wt.% NaCl solution. However, when we added trace amount of the Ag element to the Al-0.1Cu alloy (Figure 8b), the Al(OH)_3_ phase’s diffraction peaks almost vanished on the corroded Al-0.1Cu-0.1Ag alloy, which could be explained according to the Tafel test. The corrosion resistance of the Al-Cu alloy was significantly enhanced by Ag microalloying, thus the quantity of Al(OH)_3_ products generated during the electrochemical corrosion was greatly reduced.

## 4. Conclusions

In this work, the microscopic structure, mechanical characteristics and corrosion resistance performance of Al-0.1Cu alloy with different Ag content and Al-0.15Cu-0.1Ag alloy were comprehensively examined. The introduction of the Ag element promoted the formation of the θ’ phase and restrained the growth of the θ’ phase. The Al-0.1Cu-0.08Ag alloy’s tensile strength at room temperature was marginally lower than that of the Al-0.1Cu alloy, suggesting that the addition of Ag has little impact on the tensile strength of the Al-0.1Cu alloy. When the Cu content increased from 0.1 wt.% to 0.15 wt.%, the σ_0_._2_ and σ_b_ of the Al-0.15Cu-0.1Ag alloy increased, which was the result of the formation of more θ’ precipitates.

The alloy foils showed a high electrical conductivity of 58% ICAS, indicating that the electrical conductivity of the Al-Cu alloys was not significantly affected by Ag microalloying. The findings of the electrochemical testing showed that the Al-Cu alloy’s corrosion resistance was significantly improved by the addition of a moderate amount of Ag. The E_corr_ changed positively as the Ag content increased, and the I_corr_ also significantly dropped, showing that the Ag-containing alloys were more resistant to corrosion once it had started. Moderate Ag alloying with Al-Cu alloys has demonstrated promising comprehensive properties for application as current collectors of LIBs.

## Figures and Tables

**Table 1:** Mechanical characteristics of the four heat-treated alloys tested at 25 °C and 60 °C.

Temperature	Alloy Composition		σ_0.2_ (MPa)	σ_b_ (MPa)	δ (%)
	Base	Ag Addition			
25 °C	Al-0.1Cu	0	175 ± 4	189 ± 3	1.6 ± 0.1
		0.08	170 ± 5	184 ± 5	1.4 ± 0.1
		0.1	179 ± 3	196 ± 1	1.5 ± 0.1
	Al-0.15Cu	0.1	185 ± 4	205 ± 4	1.3 ± 0.1
60 °C	Al-0.1Cu	0	156 ± 2	176 ± 3	1.7 ± 0.1
		0.08	154 ± 2	172 ± 1	1.6 ± 0.1
		0.1	158 ± 2	180 ± 2	1.6 ± 0.1
	Al-0.15Cu	0.1	165 ± 3	179 ± 3	1.6 ± 0.1

**Table 2:** Electrical conductivity of the Al-xCu-yAg alloys.

Alloy	Electrical Conductivity (%IACS)
Al-0.1Cu	58.43 ± 0.21
Al-0.1Cu-0.08Ag	58.12 ± 0.17
Al-0.1Cu-0.1Ag	57.94 ± 0.12
Al-0.15Cu-0.1Ag	58.41 ± 0.19

**Table 3:** Tafel polarization parameters of the heat-treated alloy foils.

Alloys Composition (wt.%)	E_corr_ (mV vs. SCE)	I_corr_ (μA/cm^2^)
Al-0.1Cu	−873 ± 7	37.12
Al-0.1Cu-0.08Ag	−721 ± 5	2.62
Al-0.1Cu-0.1Ag	−706 ± 5	5.49
Al-0.15Cu-0.1Ag	−655 ± 6	5.70

## Data Availability

Not applicable.
